# Operational assessment of point-of-care diagnostics in rural primary healthcare clinics of KwaZulu-Natal, South Africa: a cross-sectional survey

**DOI:** 10.1186/s12913-018-3207-6

**Published:** 2018-05-29

**Authors:** T. P. Mashamba-Thompson, B. Sartorius, P. K. Drain

**Affiliations:** 1 Discipline of Public Health Medicine, School of Nursing and Public Health, University of KwaZulu-Natal, 2nd Floor, George Campbell Building, Science Drive, Howard College Campus, Durban, 4001 South Africa; 2 International Clinical Research Center, Department of Global Health, University of Washington, Seattle, USA; 3 Division of Infectious Diseases, Department of Medicine, University of Washington, Seattle, USA; 4 Department of Epidemiology, University of Washington, Seattle, USA; 5 Department of Surgery, Massachusetts General Hospital, Boston, MA USA

**Keywords:** Point-of-care test, Diagnostic testing, Primary healthcare clinics, Rural health, Health services, KwaZulu-Natal, South Africa

## Abstract

**Background:**

The World Health Organization (WHO) called for new clinical diagnostic for settings with limited access to laboratory services. Access to diagnostic testing may not be uniform in rural settings, which may result in poor access to essential healthcare services. The aim of this study is to determine the availability, current usage, and need for point-of-care (POC) diagnostic tests among rural primary healthcare (PHC) clinics in South Africa’s KwaZulu-Natal (KZN) province.

**Methods:**

We used the KZN’s Department of Health (DoH) clinic classification to identify the 232 rural PHC clinics in KZN, South Africa. We then randomly sampled 100 of 232 rural PHC clinics. Selected health clinics were surveyed between April to August 2015 to obtain clinic-level data for health-worker volume and to determine the accessibility, availability, usage and need for POC tests. Professional healthcare workers responsible for POC testing at each clinic were interviewed to assess the awareness of POC testing. Data were survey weighted and analysed using Stata 13.

**Results:**

Among 100 rural clinics, the average number of patients seen per week was 2865 ± 2231 (range 374–11,731). The average number of POC tests available and in use was 6.3 (CI: 6.2–6.5) out of a potential of 51 tests. The following POC tests were universally available in all rural clinics: urine total protein, urine leukocytes, urine nitrate, urine pregnancy, HIV antibody and blood glucose test. The average number of desired POC diagnostic tests reported by the clinical staff was estimated at 15 (CI: 13–17) per clinic. The most requested POC tests reported were serum creatinine (37%), CD4 count (37%), cholesterol (32%), tuberculosis (31%), and HIV viral load (23%).

**Conclusion:**

Several POC tests are widely available and in use at rural PHC clinics in South Africa’s KZN province. However, healthcare workers have requested additional POC tests to improve detection and management of priority disease conditions.

**Trial registration:**

Clinical Trials.gov Identifier: NCT02692274

**Electronic supplementary material:**

The online version of this article (10.1186/s12913-018-3207-6) contains supplementary material, which is available to authorized users.

## Background

Diagnostic testing is a fundamental health service in resource-limited settings [1–4]. Access to diagnostic testing may not be uniform in rural settings, which may result in poor access to essential healthcare services [5–9]. In addition, delays in diagnostic testing are a major consideration in rural communities that lack on-site laboratory infrastructure [10, 11]. The World Health Organization (WHO) called for new clinical diagnostic methods that are designed to function in settings with limited access to laboratory services [12]. Point-of-care (POC) tests have been proven to be effective for strengthening health systems by providing rapid results to improve timely initiation of suitable therapy, facilitate linkages to care, and improve health outcomes [4, 13–19]. These tests are intended to assist clinical staff in performing diagnostic testing at the clinical point-of-care [20–23]. The introduction of POC diagnostics in remote and resource-limited settings has been proven to help improve healthcare access and patient outcomes [24, 25].

There has been increased availability of POC testing in rural and resource-limited settings [1, 12]. The WHO provides guidelines for resource-limited-setting POC diagnostic services to ensure that POC diagnostics address the needs of the user in a clinically and cost effective manner [26]. Accelerating access to innovations through the implementation of POC diagnostic tests in rural, remote, resource-limited disease-burdened settings is one of the WHO’s introduced strategic health priority actions [27]. However, barriers and challenges to implementing POC diagnostics in resource-limited settings have been demonstrated [28–30].

Delayed disease diagnosis, as a consequence of poor access to healthcare services, has been reported as one of the major problems in rural communities [10]. Determining the current accessibility, availability and utility of POC diagnostics in these settings is vital to ensure successful implementation and sustainability of new POC testing. Despite this, to date, the level of POC diagnostic accessibility, availability, usage and need in South African primary care clinics has not been evaluated. We aimed to determine the current accessibility, availability, usage, and future needs for POC tests throughout the rural primary healthcare (PHC) clinics of KwaZulu-Natal (KZN), South Africa.

## Methods

### Data sources and procedures

We conducted a cross-sectional survey of rural PHC clinics in the KZN province from April to August 2015. We used the KZN’s Department of Health (DoH) clinic classification to identify the 232 rural PHC clinics. We used DoH estimates for the population size currently served by each PHC clinic. We then randomly selected a weight-based sample of 100 rural PHC clinics from all 11 KZN districts to ensure uniform sampling across districts, as described in our protocol [31]. We obtained an average daily patient census from the 2014 South African District Health Information Software (DHIS) to stratify the 100 clinics into four proportionate strata. Proportionate stratification was implemented to ensure that sample size of each stratum is proportionate to the population size of the strata amongst all 11 KZN districts. Using the PHC average daily patient census data reported by DHIS, a proportionate stratification of 100 primary sampling units (PSU) across the four strata was utilised Each stratum consisted of 25 facilities randomly selected by probability proportional to size (PPS). Sample size was checked against the size variable, which was used in PPS sampling. Comparison of the projected number of rural PHC clinics by strata and district, based on the applying the sample weights, suggested that the sample was representative [31].

We developed a POC diagnostics survey questionnaire with guidance from Howick et al. 2014 survey tool and the World Organization of Family Doctors (WONCA) special interest group for global POC testing online survey [32, 33], we conducted a pilot test of the survey in five rural PHC clinics in KZN, and adjusted the survey tool based on feedback from respondents. The final survey questionnaire consisted of closed-ended questions suitable for the local context (Additional file 1).

We surveyed each sampled clinic to record the number of staff nurses, professional nurses, PHC specialist nurses, and operations managers, as well as their years of experience. We assessed accessibility and availability of POC diagnostics in rural PHC clinics through a questionnaire and interviews with PHC healthcare professionals who are responsible for the POC diagnostic services in the clinic. To determine the PHC clinic characteristics, we obtained data on the average number of patients and nurses for each clinic from the DHIS. Data on POC diagnostic accessibility and availability were ascertained from the nurses’ responses to questions used to measure the accessibility and availability of POC diagnostic tests using the survey tool. In order to determine the level of usage, the responses that indicated POC test usage were followed up by a question on frequency of test usage with responses for ‘more than once per day’ , ‘daily’ , ‘weekly’ , ‘monthly’ and ‘once per year or less’. Response on frequency of use was analysed using a 1–5 score scale, where ‘5 = more than once per day’ , ‘4 = daily’ , ‘3 = weekly’ , ‘2 = monthly’ and ‘1 = once per year or less’. Therefore, POC test usage frequency score ranged from one to five, one being the lowest and five being the highest.

From each clinic, we obtained additional information on distance to the closest town and emergency department. To ensure accurate estimation of the distance between the sampled PHC clinic and emergency hospitals or cities/towns, we obtained the health facility geographic information system (GIS) coordinates from the KZN Department of Health database. The sampled PHC clinic GIS coordinates were uploaded and analysed using Google maps to generate accurate estimates of travelling distances. One respondent was interviewed from each clinic from healthcare workers responsible for POC diagnostics services. Respondents’ level of POC diagnostic test awareness was determined by the three open ended questions used to measure a health worker’s awareness of POC diagnostic tests (Additional file 1). Responses on the level of awareness were analysed in percentage scores. A response of five correct tests was considered as high awareness, whereas a score lower than 3 was considered poor awareness and a score between 3 and 4 was considered average awareness. Awareness scores were calculated in percentages by summing the total number of correct answers with a highest score indication. Responses on health workers’ POC test awareness were analysed using a 0–100% score scale, where ‘0% = 0 test’ , ‘20% =1 test’ , ‘40% = 2 tests’ , ‘60% = 3 tests’ , ‘80% = 4 tests’ and ‘100% = 5 tests’. To determine the most desirable or needed POC tests, respondents were asked to select tests in our survey sheet and to list conditions in which they would like POC tests to help them make diagnoses and make their work easier. Participants’ responses regarding the conditions that they would like in order for POC diagnostics to help them make a diagnosis were categorised into either communicable or non-communicable diseases. Some POC tests, such as creatinine, were included for both communicable and non-communicable diseases.

### Outcomes

The primary outcome of the study was to assess the accessibility and availability of individual POC diagnostics in rural PHC clinics in KZN, South Africa. The secondary outcome of this study was to determine the current need for POC diagnostics in rural PHC clinics in KZN, South Africa.

### Statistics analyses

The survey weight calculation has been described [31]. Data were processed and analysed using Stata Statistical Software, Release 13. (College Station, TX: StataCorp LP). The PHC clinics data and respondent characteristics were compared and analysed with characteristics of the PHC clinic obtained from DHIS. We applied survey weights given sampling design to construct 95% confidence intervals. Stata (version 13) was employed for generation of response frequencies. Details on frequently of use of POC diagnostics and the level of need for tests are displayed in tables.

## Results

The survey received a 100% response rate from randomly selected rural PHC clinics and staff in rural KwaZulu-Natal. Table 1 presents the characteristics of the sampled rural PHC clinics. Overall, 40 (40%) of the sampled clinics were located within 10 km of an emergency tertiary hospital. The average number of patients using the sampled rural PHC clinics per week was estimated at 2864.9 ± 2230.9 (range 374–1117.3). The staff profile for survey respondents was as follows: 23% operations managers, 76% PHC specialist nurses, and 1% staff nurses.

**Table 1:** Description of 100 rural PHC clinics surveyed in KwaZulu-Natal

Variable	Mean and standard deviation	Range
Healthcare Workers		
Respondent year of qualification	1999 ± 9	1976–2015
Number of Doctors	0.1 ± 0.3	0–2
Number of Community Health Workers	12.4 ± 11.8	0–65
Number of HIV councillors	1.2 ± 1.	0–3
Number of Nurses	10.3 ± 5.6	2–38
Clinical Data		
PHC weekly patient census	2865 ± 2231	374–11,731
PHC number of nurses (DHIS 2014)	25 ± 23	2–120
Distance from the nearest hospital (kilometres)	41.4 ± 42.8	0.1–278.0
Distance from the nearest town (kilometres)	45.0 ± 50.6	0·02–228
POC diagnostics in use	63.9 ± 0.8	5–8
POC diagnostics needed	14.2 ± 9.9	0–51
Respondents’ Position (%)		
Operations Manager	23	23%
Specialist Nurse	76	76%
Staff Nurse	1	1%

The PHC clinic with the lowest and highest number of PHC clinic nurses was reported in Mbhugwini clinic with two nurses and Thokozani clinic with 38 nurses, respectively. The lowest and highest average PHC clinic patient consensus was reported in Thokozani and Mabibi clinic, respectively. All respondents (nurses), reported working standard working hours of 40 h per week. The audited average number of nurses reported in the sampled clinics was 10.3 ± 5.6. The audited number of professional staff members employed in rural PHC suggest that community health workers form the largest staff complement in PHC clinics, with an average of 12.6 ± 11.8 per clinic, followed by nurses 10.3 ± 5.6, lay councillors, 1.2 ± 1.0 and doctors, 0.1 ± 0.3, respectively.

**Fig. 1:**
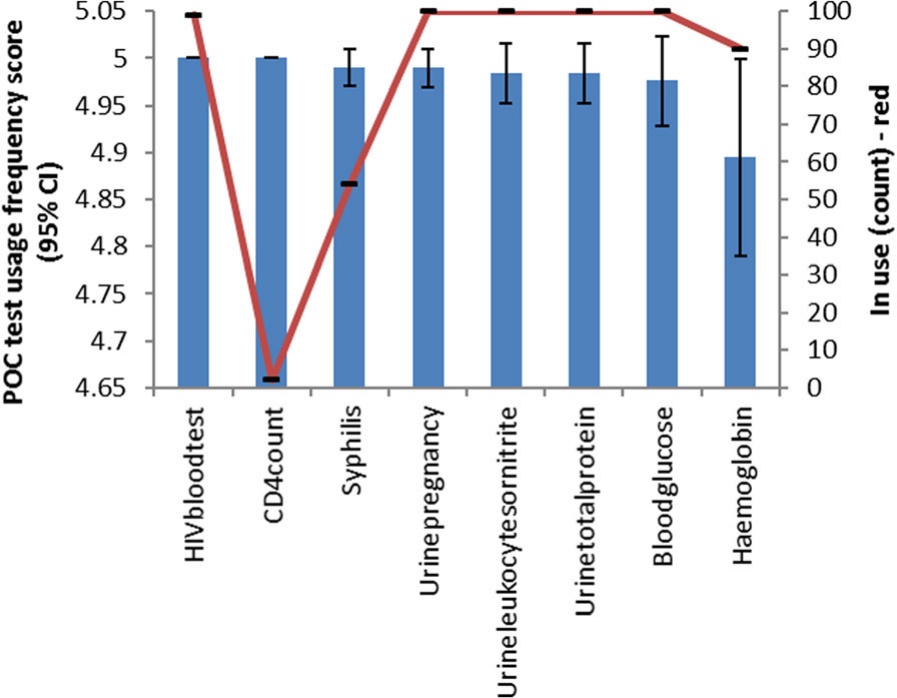
Point-of-care tests availability for use and frequency of use in rural KwaZulu-Natal PHC clinics

On average in each surveyed clinic, out of the currently available tests listed in on the survey sheet, an average of 6.4 ± 0.8 tests was available for use (Table 1). All clinics employed the following POC tests: HIV rapid test (RT), total protein (urine), leukocytes or nitrate (urine), pregnancy (urine) and blood glucose POC tests. Amongst the eight used POC tests, the following tests were reported as the most frequently used tests in KZN rural PHC clinics, with an estimated usage frequency score of 5.0 (CI: 5.0–5.0): blood glucose, HIV RT and urine pregnancy, urine leukocytes and nitrite, urine protein and CD4 count (Fig. 1). Although CD4 count POC test was reported as the one of the most frequently used POC tests in KZN rural PHCs, it was only available in two clinics.

The average POC awareness score amongst the interviewed healthcare professionals for POC used for diagnosing disease, POC used for monitoring and managing disease and POC used for reducing referrals was estimated at 4.1 (CI: 3.8–4.3); 3.0 (CI: 2.7–3.4); and 2.3 (CI: 1.9–2.6), respectively (Table 2). Survey results show different levels of POC diagnostic accessibility, availability and frequency of use amongst the sampled PHC clinics. Table 3 displays an average number of POC diagnostic tests that are currently accessible, available and in use in the sampled rural PHC clinics.

**Table 2:** Category of respondents and prevalence of point-of-care diagnostic awareness in rural KwaZulu-Natal primary healthcare clinics

POC test awareness	Average awareness score out of 5 and 95% confidence interval (CI)	Range
Awareness of POC tests used for diagnosing disease	4.1 (CI: 3.8–4.3)	0–5
Awareness of POC tests used for monitoring and management of disease	3.0 (CI: 2.7–3.4)	0–5
Awareness of POC tests used for reducing referrals to secondary and tertiary health institution	2.3 (CI: 1.9–2.6)	0–5

**Table 3:** Availability and usage of point-of-care tests in rural KwaZulu-Natal primary healthcare clinics

POC test	Average number of clinics using the test (95% CI)	Frequency of use ranging from 1 to 5 (95% CI)
Blood glucose	1.0 (−)	5.0 (−)
Haemoglobin	0.9 (CI: 1.0–0.8)	4.2 (CI: 3.8–4.6)
HIV rapid test	1.0(−)	5.0 (−)
Syphilis	0.5 (CI: 0.6–0.4)	4.07 (CI: 3.5–4.6)
Urine pregnancy	1.0 (−)	5.0 (−)
Urine leukocytes and nitrite	1.0 (−)	5.0 (−)
Urine protein	1.0 (−)	5.0 (−)
CD4 count	0.02 (CI: 0.1–0)	5.0 (−)

**Fig. 2:**
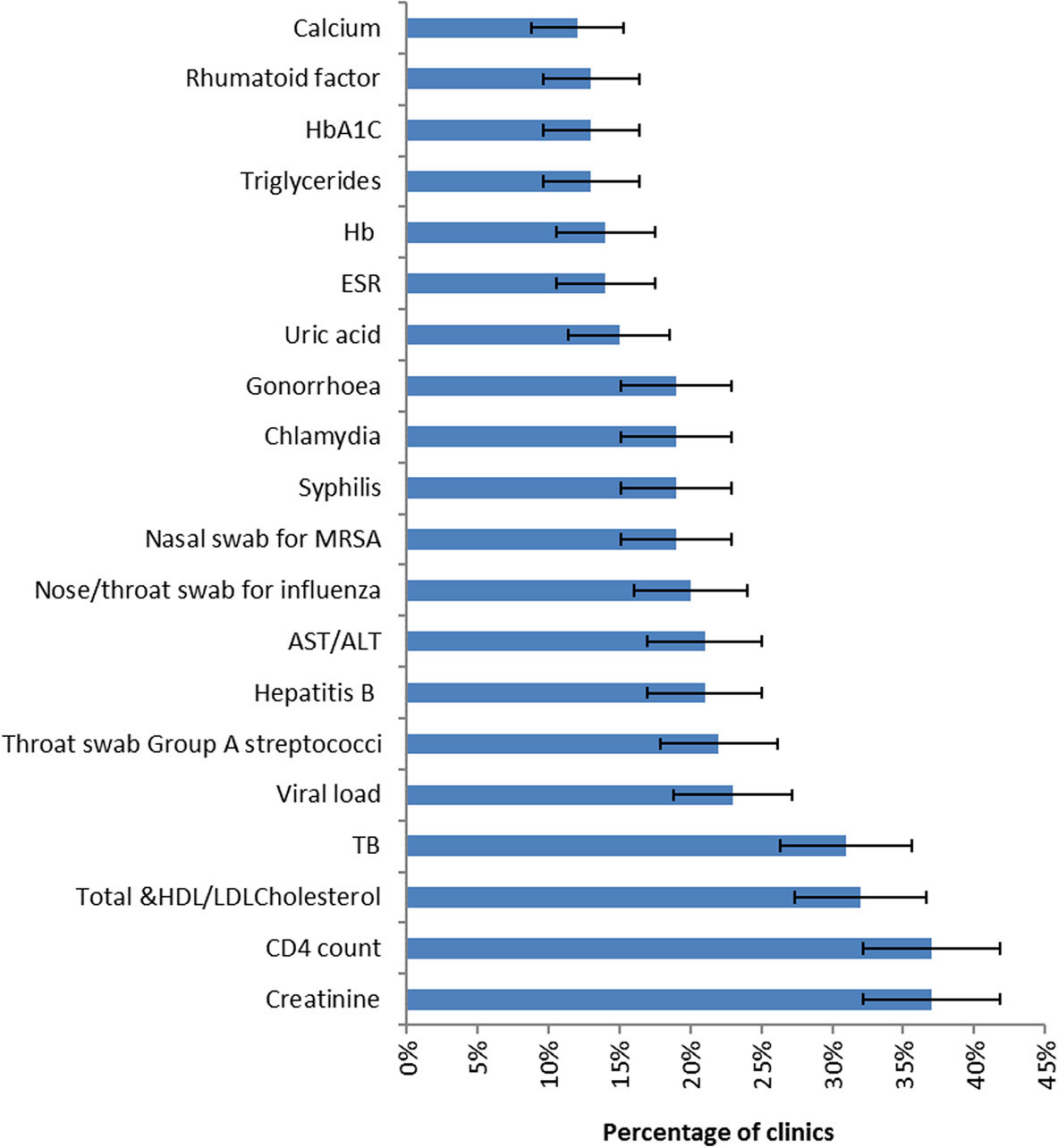
Top 20 requested point-of-care tests in rural KwaZulu-Natal primary healthcare clinics

The survey results show that desire for future use of POC tests was higher than the current use. PHC nurses in rural KZN reported a need for 80 tests at POC in comparison to the average of 6.4 ± 0.8 available tests (Fig. 2. Table 4 shows the top 10 of the most desirable tests by test class and disease category. Amongst the suggested or desirable POC test disease targets, 54% were for non-communicable diseases, 35% were for communicable diseases, and the remaining 11% were for both communicable and non-communicable diseases. Total cholesterol and HDL/LDL cholesterol were reported as the most desired targets for POC tests amongst the non-communicable disease group, as suggested by 32% of the clinics. Of the communicable disease group, the CD4 count POC test was the most desirable, requested by 37% of the clinics.

**Table 4:** List of desirable point-of-care tests by test class and disease type: top 10 for each category

Communicable diseases (*n* = 29)		Non-communicable disease (*n* = 45)		Communicable and non-communicable (*n* = 6)	
*Condition*	*N (%)*	*Condition*	*N (%)*	*Condition*	*N (%)*
CD4 count	37 (37%)	Total & HDL/LDL Cholesterol	32 (32%)	Creatinine	37 (37%)
TB	31 (31%)	ESR	14 (14%)	X-ray	5 (5%)
Viral load	23 (23%)	Triglycerides	13 (13%)	Creatinine reactive protein (CRP)	2 (2%)
Hepatitis B	21 (21%)	HbA1C	13 (13%)	Vision test	2 (2%)
AST/ALT	21 (21%)	Rhumatoid factor	13 (13%)	Hearing test	1 (1%)
Nose/throat swab for influenza	20 (20%)	Calcium	12 (12%)	Eye testing	1 (1%)
Nasal swab for MRSA	19 (19%)	TSH	11 (11%)	Foot care	1 (1%)
Syphilis	19 (19%)	Potassium	11 (11%)	n/a	n/a
Chlamydia	19 (19%)	White cell count	11 (11%)	n/a	n/a
Gonorrhoea	19 (19%)	Sodium	10 (10%)	n/a	n/a

N, percentage of clinics

## Discussion

In South Africa, the challenge of healthcare provision in rural areas is largely linked to the “physical, demographic, economic, social and cultural context” that characterises rural settings [34]. This study revealed poor POC diagnostic accessibility, which has an impact on availability and usage. It was demonstrated that a high number of POC tests is needed to assist PHC clinic healthcare workers with decisions, such as for immediate treatment and urgent referrals. POC tests to assess kidney function, presence of a urinary tract infection, pregnancy status, HIV status, and blood glucose were widely available and regularly used in rural clinics throughout the KwaZulu-Natal province. Of the available POC tests, the CD4 count POC test were available in two clinics, but were reported as being among the most frequently used tests, with a frequency of use at more than once per day. These findings are in line with the recently reported on-going high HIV prevalence in rural KZN [35]. The results of this study also suggest a POC diagnostic knowledge gap amongst PHC clinic nurses, demonstrated by their poor awareness of POC tests used for monitoring and for reducing referrals.

Stratification of representative sample size was conducted in this study to reduce sampling error and ensure generalisation of the results to rural PHC clinics in South Africa. The results of this study demonstrated the current stance on POC diagnostic accessibility, availability and usage, as well as the need for future POC diagnostics from the healthcare workers’ perspective. The study reports CD4 count, creatinine and cholesterol as the most needed tests, and this is in line with the current local double disease burden due to HIV and non-communicable diseases [36, 37]. Knowledge of local epidemiology and diagnostic accessibility are some of the key factors during implementation of new diagnostics [38]. Although the study has provided important information relating to contextual needs for POC diagnostics in high HIV-prevalence and resource-limited settings, these findings cannot be generalised to the implementation of POC diagnostics in a low HIV-prevalence and developed setting. The causes of poor accessibility, availability and use for POC diagnostics were not determined in this study. A parallel study was conducted to determine the reasons for these deficiencies from multiple POC diagnostic key stakeholders’ perspectives.

The current survey results refute previous findings from POC diagnostic surveys conducted in the developed world PHC clinics [39, 40]. The United States (US) survey by Sohn et al. study aimed at assessing the use of POC diagnostics and the perceived benefits, as well as concerns regarding POC diagnostics among family physicians in the have shown that the following are the top three disease conditions for which physicians reported using POC tests for diagnosis: diabetes mellitus, urinary tract infections and strep throat [40]. The United Kingdom (UK) survey by Turner et al. aimed at establishing conditions for which POC tests would be most helpful for diagnosis, has shown that GPs find POC tests most useful for the following disease conditions: urinary tract infections, pulmonary embolism/deep vein thrombosis and international normalized ratio/anticoagulation [39]. However, A study was conducted by Howick et al. 2014, which used a similar survey tool to determine which POC tests healthcare workers in the UK, USA, Australia, Belgium and the Netherlands are currently using or would like to use [32] demonstrated a high need for HIV-related POC tests. These results demonstrated the need for assessment of each context to ensure appropriate implementation of POC diagnostics to meet patients’ needs.

Although this study enabled the determination of the current and future need for POC diagnostics in rural PHC clinics in KZN, the method of data collection used limited the ability to determine the reasons for deficiencies in POC availability in some of the clinics. In addition, although clinicians were requested to provide a list of tests needed in their clinics in response to their local health problems, the survey tool did not enable them to provide the frequency of use for some of the requested tests. The survey too also did not include respondent’s age. Therefore, we are unable to tie the respondent’s age with their years of experience. This information is useful, as it can guide implementers on the most useful interventions and POC tests to prioritise during the implementation of new tests and to ensure sustainability of these services. One unavoidable limitation of this study was the selection of one survey respondent from each participating clinic, therefore responses were based on one respondent’s perception of the current availability and use as well as future needs of POC diagnostics in the participating clinic.

The results of this study provided an overview of local disease burden and of diseases which should be prioritised during implementation of future POC diagnostics. The WHO recently (2017) proposed an essential diagnostics lists for resource-limited settings [41]. The results of this study demonstrate poor accessibility and availability of the essential diagnostics recommended by the WHO. Poor access to diagnostic services for infectious diseases is a serious impediment to improving the health of a nation [42], particularly in high HIV pandemic regions. Fast-tracking the HIV/AIDS response to increase the number of people on treatment and to achieve the UNAIDS 90–90-90 targets by 2020 is one of the global health priorities [43]. Therefore, urgent supply of these essential diagnostics is required to address the unmet needs of patients in rural and resource-limited settings. Successful supply or deployment of essential diagnostics to the rural and resource-limited settings will require adequate supply chain management (SCM). We recommended a lean and agile quality systems management for low-and-middle-income countries (LMICs) to help ensure adequate supply chain management and sustainability POC diagnostics services in these settings [44]. The results also reveal a high level of professional nurse shortage. In addition, a POC diagnostic knowledge gap amongst PHC clinic nurses was demonstrated by nurses’ poor awareness of POC diagnostics that are used for monitoring and for reducing referrals. Adequate resources and appropriate training of staff has been demonstrated as critical in ensuring the suitable administration of diagnostic tests [45]. We have recommended a POC diagnostics training program to help improve staff performance and ensure continual quality improvement for POC diagnostics in these settings [46]. Efforts to improve the rapport between diagnostic developers, decision makers, implementers and users (healthcare workers) are recommended to improve accessibility, availability and usage of POC diagnostics for improvement of health outcomes in rural and resource-limited settings.

Furthermore, the results of this study can help inform developers and implementers of POC diagnostics on context-specific implementation of POC diagnostics to address the unmet needs of patients in these settings. Bearing in mind the current disease burden in rural KwaZulu-Natal, improved access to the users’ most desirable POC diagnostics is recommended, particularly in remote communities with limited laboratory infrastructure. Further research to assess the role of the users’ most desirable diagnostic tests is highly recommended. Efforts to ensure appropriate implementation of future POC diagnostics are urgently needed from relevant stakeholders, including researchers, laboratory managers, POC diagnostics developers, policy makers and users.

## Conclusion

This study has shown the need for scaling up POC tests in rural PHC clinics in KwaZulu-Natal, South Africa, from users’ perspective. Interventions should ensure that adequate human resources are emplaced for the suitable administration of the POC test and the correct interpretation of the test result, prior to test implementation. Future studies should assess the utility and impact of currently used POC diagnostics on health outcomes in order to further justify the need for scale up.

## Additional file

Additional file 1: Survey tool for POC diagnostic in rural KwaZulu-Natal, adapted from Howick et al. 2014, and the World Organization of Family Doctors (WONCA) special interest group for global point-of-care testing online survey. (DOCX 29 kb)

## Abbreviations

AIDS: Acquired Immunodeficiency Syndrome
ART: Antiretroviral treatment
ASSURRED: Affordability, sensitivity, specificity, user friendly, rapid and robust, equipment free and delivered
CD4^+^: Cluster of differentiation 4
CI: Confidence intervals
DHIS: District Health Information System
DoH: Department of Health
HIV: Human Immunodeficiency Virus
KZN: KwaZulu-Natal
LMIC: Low and middle income countries
MDG: Millennium Development Goal
PHC: Primary Healthcare
PMTCT: Preventing mother-to-child transmission
POC: Point of Care
POCT: Point of care test/testing
SDG: Sustainable Development Goals
TB: Tuberculosis
VL: Viral load
WHO: World Health Organization
