# Supplementary material for: Operational assessment of point-of-care diagnostics in rural primary healthcare clinics of KwaZulu-Natal, South Africa: a cross-sectional survey
Source: BMC Health Serv Res. 2018 May 29;18:380. doi: 10.1186/s12913-018-3207-6 (PMC5975682; doi:10.1186/s12913-018-3207-6)
Supplement: Supplementary file 1 — Survey tool for POC diagnostic in rural KwaZulu-Natal, adapted from Howick et al. 2014, and the World Organization of Family Doctors (WONCA) special interest group for global point-of-care testing online survey. (DOCX 29 kb) [file 12913_2018_3207_MOESM1_ESM.docx]

**Survey tool**

| ***University of KwaZulu Natal, Durban, School of Nursing and Public Health, Discipline of Public Health Medicine***  ***Title:*** *Evaluating the accessibility and utility of HIV-related point of care (POC) diagnostics for maternal health in rural South Africa* |
| --- |

**PHC POC Diagnostics Services Survey tool**

| **District code:** | **Clinic code:** |
| --- | --- |
| **District name:** | **Clinic name:** |
| **Assessors name:** | **Date:** |

| **A** | **Clinic size and staffing** | |
| --- | --- | --- |
| A1 | Which of the following healthcare professional are in your clinic? | *(Choose from list below)*   - Drs: number___________ - Nurses: Number_________ - Specialist Nurse__________ - Sisters_______________ - Other (Specify)____________ |
| A2 | Which of the following describe your role? | *(Choose from list below)*   - Dr - Nurse - Specialist Nurse - Sister - Other (Specify)____________ |
| A3 | What is an average number of MH patients admitted in the clinic per week? | *(Write number)* |
| A4 | How many hours per week do you work (on average)? | *(Write number)* |
| **B** | **POC diagnostics service linkage to healthcare** |  |
| B1 | How many kilometers to your nearest emergency department that admits patients to hospital? | *(Write number)* |
| B2 | How many kilometers to your nearest town/city? | *(Write number)* |
| **C** | **Length of time for blood test** |  |
| C1 | How long does it typically take to get results from a routine blood test, such as full blood count? | *(Choose from the following)*   - One day or more: _______days - Less than 1 day: _______ hours - Already use a POC diagnostics for this test, so it is immediately done |
| **D** | **Staff POC diagnostics competency** |  |
| D1 | What year did you qualify as a Healthcare professional? | *(Write full year)* |
| D2 | Please name up to five conditions which a POC test could help you make a **diagnosis**. Please list the conditions irrespective of whether or not POC test currently exist | - ____________________ - _____________________ - _____________________ - _____________________ - _____________________   **I do not think POC tests would help me make a diagnosis (please tick box)** |
| D3 | Please name up to five conditions which a POC test could help you make a **monitor**. Please list the conditions irrespective of whether or not POC test currently exist | - ____________________ - _____________________ - _____________________ - _____________________ - _____________________   **I do not think POC tests would help me monitor a disease (please tick box)** |
| D4 | Please name up to five conditions which a POC test could help you make a **reducing referrals for specialty care or hospital admission**. Please list the conditions irrespective of whether or not POC test currently exist | - ____________________ - _____________________ - _____________________ - _____________________ - _____________________   **I do not think POC tests would help me reduce referrals for specialty care or hospital admission (please box)** |

| **E. POCTs used**  *Please select the answer that best matches your views about current or potential use of point of care tests (POCTs)* | | | | |
| --- | --- | --- | --- | --- |
|  | This test **is** currently available as a point of care test (POCT) in my clinic | | This test **is not** currently available as a point of care test (POCT) in my clinic | |
|  | *(1)* I **do** use this test | *(2)* I **do not** use this test | *(3)* I **would** use this test | *(4)* I **would not** use this test |
| **TESTS ON BLOOD** |  |  |  |  |
| **Cardiovascular** |  |  |  |  |
| Creatinine |  |  |  |  |
| Potassium |  |  |  |  |
| Sodium |  |  |  |  |
| Total cholesterol |  |  |  |  |
| HDL/LDL cholesterols |  |  |  |  |
| Triglycerides |  |  |  |  |
| Calcium |  |  |  |  |
| Uric Acid |  |  |  |  |
| BNP (B-natriuretic peptide) |  |  |  |  |
| D-dimer |  |  |  |  |
| Troponin |  |  |  |  |
| **Endocrine** |  |  |  |  |
| Blood glucose |  |  |  |  |
| HbA1c |  |  |  |  |
| TSH (thyroid stimulating hormone) |  |  |  |  |
| Free T4 or T3 |  |  |  |  |
| **Haematology** |  |  |  |  |
| INR |  |  |  |  |
| Haemoglobin |  |  |  |  |
| White cell count |  |  |  |  |
| Platelet count |  |  |  |  |
| Prothrombin time |  |  |  |  |
| **Infection related** |  |  |  |  |
| CRP (C-reactive protein) |  |  |  |  |
| Procalcitonin |  |  |  |  |
| HIV blood test |  |  |  |  |
| Syphilis |  |  |  |  |
| Hepatitis B |  |  |  |  |
| **Liver** |  |  |  |  |
| AST/ALT |  |  |  |  |
| Alkaline phosphatase |  |  |  |  |
| Bilirubin |  |  |  |  |
| Gamma GT (ɣ-glutamyltransferase) |  |  |  |  |
| Albumin |  |  |  |  |
| **Other (blood)** |  |  |  |  |
| ESR (*Erythrocyte sedimentation rate)* |  |  |  |  |
| CA125 |  |  |  |  |
| PSA (Prostate Specific Antigen) |  |  |  |  |
| Vitamin D |  |  |  |  |
| Vitamin B12 |  |  |  |  |
| Folate |  |  |  |  |
| Quantitative Beta HCG (Human chorionic gonadotropin) |  |  |  |  |
| Rheumatoid factor |  |  |  |  |
| ANA (anti-nuclear antibodies) |  |  |  |  |

|  | This test **is** currently available as a point of care test (POCT) in my clinic | | This test **is not** currently available as a point of care test (POCT) in my clinic | |
| --- | --- | --- | --- | --- |
|  | *(1)* I **do** use this test | *(2)* I **do not** use this test | *(3)* I **would** use this test | *(4)* I **would not** use this test |
| **RESPIRATORY SAMPLES** |  |  |  |  |
| Throat swab for Group A Streptococci |  |  |  |  |
| Nasal swab for MRSA |  |  |  |  |
| Nose/throat swab for influenza |  |  |  |  |
| **TESTS ON URINE OR GENITAL FLUIDS** |  |  |  |  |
| Urine pregnancy test |  |  |  |  |
| Urine leukocytes or nitrite |  |  |  |  |
| Chlamydia |  |  |  |  |
| Gonorrhoea |  |  |  |  |
| Urine albumin:creatinine ratio |  |  |  |  |
| Urine total protein |  |  |  |  |
| Urine protein:creatinine ratio |  |  |  |  |
| **TESTS ON FAECES** |  |  |  |  |
| Faecal occult blood |  |  |  |  |
| Faecal calprotectin |  |  |  |  |
| **OTHER TESTS WE HAVE NOT LISTED HERE** |  |  |  |  |

| **F. Frequency of POCT usage**  *Below is a list of point of care tests (POCTS) you indicated that you would use or currently use in your practice. Please tell us how often you would use or do use these* | | | | | |
| --- | --- | --- | --- | --- | --- |
|  | More than once per day | Daily | Weekly | Monthly | Once per year or less |
| **TESTS ON BLOOD** |  |  |  |  |  |
| **Cardiovascular** |  |  |  |  |  |
| Creatinine |  |  |  |  |  |
| Potassium |  |  |  |  |  |
| Sodium |  |  |  |  |  |
| Total cholesterol |  |  |  |  |  |
| HDL/LDL cholesterols |  |  |  |  |  |
| Triglycerides |  |  |  |  |  |
| Calcium |  |  |  |  |  |
| Uric Acid |  |  |  |  |  |
| BNP (B-natriuretic peptide) |  |  |  |  |  |
| D-dimer |  |  |  |  |  |
| Troponin |  |  |  |  |  |
| **Endocrine** |  |  |  |  |  |
| Blood glucose |  |  |  |  |  |
| HbA1c |  |  |  |  |  |
| TSH (thyroid stimulating hormone) |  |  |  |  |  |
| Free T4 or T3 |  |  |  |  |  |
| **Haematology** |  |  |  |  |  |
| INR |  |  |  |  |  |
| Haemoglobin |  |  |  |  |  |
| White cell count |  |  |  |  |  |
| Platelet count |  |  |  |  |  |
| Prothrombin time |  |  |  |  |  |
| **Infection related** |  |  |  |  |  |
| CRP (C-reactive protein) |  |  |  |  |  |
| Procalcitonin |  |  |  |  |  |
| HIV blood test |  |  |  |  |  |
| Syphilis |  |  |  |  |  |
| Hepatitis B |  |  |  |  |  |
| **Liver** |  |  |  |  |  |
| AST/ALT |  |  |  |  |  |
| Alkaline phosphatase |  |  |  |  |  |
| Bilirubin |  |  |  |  |  |
| Gamma GT (ɣ-glutamyltransferase) |  |  |  |  |  |
| Albumin |  |  |  |  |  |
| **Other (blood)** |  |  |  |  |  |
| ESR (*Erythrocyte sedimentation rate)* |  |  |  |  |  |
| CA125 |  |  |  |  |  |
| PSA (Prostate Specific Antigen) |  |  |  |  |  |
| Vitamin D |  |  |  |  |  |
| Vitamin B12 |  |  |  |  |  |
| Folate |  |  |  |  |  |
| Quantitative Beta HCG (Human chorionic gonadotropin) |  |  |  |  |  |
| Rheumatoid factor |  |  |  |  |  |
| ANA (anti-nuclear antibodies) |  |  |  |  |  |

|  | More than once per day | Daily | Weekly | Monthly | Once per year or less |
| --- | --- | --- | --- | --- | --- |
| **RESPIRATORY SAMPLES** |  |  |  |  |  |
| Throat swab for Group A Streptococci |  |  |  |  |  |
| Nasal swab for MRSA |  |  |  |  |  |
| Nose/throat swab for influenza |  |  |  |  |  |
| **TESTS ON URINE OR GENITAL FLUIDS** |  |  |  |  |  |
| Urine pregnancy test |  |  |  |  |  |
| Urine leukocytes or nitrite |  |  |  |  |  |
| Chlamydia |  |  |  |  |  |
| Gonorrhoea |  |  |  |  |  |
| Urine albumin:creatinine ratio |  |  |  |  |  |
| Urine total protein |  |  |  |  |  |
| Urine protein:creatinine ratio |  |  |  |  |  |
| **TESTS ON FAECES** |  |  |  |  |  |
| Faecal occult blood |  |  |  |  |  |
| Faecal calprotectin |  |  |  |  |  |
| **OTHER TESTS WE HAVE NOT LISTED HERE** |  |  |  |  |  |

**Do you have suggestions for new POC Tests?**

**What POC tests might make your job easier?**
